# Association of High Blood Pressure with Renal Insufficiency: Role of Albuminuria, from NHANES, 1999–2006

**DOI:** 10.1371/journal.pone.0037837

**Published:** 2012-07-03

**Authors:** Ping Yan, Xiangzhu Zhu, Haiming Li, Martha J. Shrubsole, Haiming Shi, Ming-zhi Zhang, Raymond C. Harris, Chuan-Ming Hao, Qi Dai

**Affiliations:** 1 Department of Cardiology, Huashan Hospital, Fudan University, Shanghai, People’s Republic of China; 2 Department of Nephrology, Huashan Hospital, Fudan University, Shanghai, People’s Republic of China; 3 Department of Medicine, Vanderbilt Epidemiology Center, Vanderbilt University School of Medicine, Nashville, Tennessee, United States of America; 4 Department of Medicine/Division of Nephrology, Vanderbilt University, Nashville, Tennessee, United States of America; Innsbruck Medical University, Austria

## Abstract

**Background:**

The relationship between hypertension and kidney disease is complicated. Clinical trials found intense blood pressure control was not associated with alterations in glomerular filtration rate (GFR) in all patients but did slow the rate of GFR decline among those with a higher baseline proteinuria. However, the underlying mechanism has been unclear.

**Methods:**

We tested the hypothesis that the association between high blood pressure and renal function is modified by albuminuria status by conducting analyses in a cross-sectional study with 12,440 adult participants without known kidney diseases, diabetes or cardiovascular diseases, participating in the National Health and Nutrition Examination Survey (NHANES) 1999–2006.

**Results:**

1226 out of 12440 were found to have unknown high blood pressure and 4494 were found to have reduced renal function. Overall, a moderate association was found between high blood pressure and renal function insufficiency in all participants analyzed. However, among participants with albuminuria, the prevalence of moderate-severe renal insufficiency substantially and progressively increased from normal subjects to prehypertensive and undiagnosed hypertensive subjects (1.43%, 3.44%, 10.96%, respectively, *P* for trend<0.0001); on the other hand, the prevalence of undiagnosed hypertension was also significantly higher among subjects with moderate-severe renal insufficiency than those with mild renal insufficiency (35.54% Vs 19.09%, *P* value <0.05), supporting an association between hypertension and renal function damage. In contrast, no association between hypertension and renal insufficiency was observed among those without albuminuria in this population. Similar findings were observed when the CKD-EPI equation was used.

**Conclusions:**

The association between high blood pressure and reduced renal function could be dependent upon the albuminuria status. This finding may provide a possible explanation for results observed in clinical trials of intensive blood pressure control. Further studies are warranted to confirm our findings.

## Introduction

Hypertension and chronic kidney disease (CKD) represent two major public health problems in the United States, both of which are linked to high risks of cardiovascular diseases [1]–[3]. According to the National Health and Nutrition Examination Survey (NHANES) data, the US prevalence for hypertension, mildly reduced kidney function (glomerular filtration (GFR) 60 to 89 mL/min/1.73 m^2^) and stage 3–4 CKD (GFR 15 to 59 mL/min/1.73 m^2^), are increasing from 24.4%, 42.4%, and 5.63% during 1988 through 1994, to 28.9%, 51.2%, and 8.04% during 1999 through 2004, respectively [4], [5].

Strong evidence indicates that treatment of hypertension not only reduces the risk of cardiovascular diseases, but also delays the progression of CKD [6]–[8]. Recently, it has been demonstrated that even having prehypertension or the earliest stages of CKD (stage 1–2) is associated with an increased risk of cardiovascular diseases [2], [9], [10]. Thus, adequate blood pressure control appears to be critical for the prevention of cardiovascular diseases and progression of CKD. However, to what extent blood pressure should be controlled is still controversial. Recently, the Accord-BP study showed that intensively targeting a systolic blood pressure of less than 120 mm Hg, as compared with less than 140 mm Hg, did not reduce the rate of a composite outcome of fatal and nonfatal major cardiovascular events in patients with type 2 diabetes at high risk for cardiovascular events [11]. Also two previous large randomized clinical trials, including the Modification of Diet in Renal Disease (MDRD) trial and the African American Study of Kidney Disease and Hypertension (AASK) trial, have failed to find a significant relationship between intense blood pressure control and glomerular filtration rate (GFR) decline among CKD patients [12]–[14]. However, in secondary analyses, progression of CKD among those with a higher baseline proteinuria was significantly delayed in the MDRD trial and a similar favorable trend was also shown in the AASK trial [15]. Very recently, the long-term follow-up study of the AASK trial further supported this view among patients with higher proteinuria [16]. These findings indicate that the association between hypertension and CKD is complicated. In this study, we tested our hypothesis that the association between high blood pressure and renal function is modified by albuminuria status. To minimize the potential influence of medication use and/or diet change on blood pressure, urinary albumin excretion or renal function, we excluded participants with self-reported kidney diseases, diabetes or cardiovascular diseases in the analyses, using data from the National Health and Nutrition Examination Survey (NHANES) 1999–2006, a population-based study conducted in the general US population.

## Results

Among the 12,440 participants who had no known cardiovascular diseases or CKD, 4,717(37.9%) of them had normal blood pressure, 3,326 (26.7%) had prehypertension, 1,226 (9.9%) had undiagnosed hypertension, and 3171(25.5%) had diagnosed hypertension. Participant characteristics by blood pressure (BP) status are shown in Table 1. 57.7% of those with normal BP were female, whereas 62.3% of prehypertensive subjects, 54.7% of undiagnosed hypertensive subjects, and 46.2% of diagnosed hypertensive subjects were male (*P* value <0.0001). Participants with abnormal BP (prehypertension or hypertension) were more likely to be older, non-Hispanic, obese (BMI≥30 kg/m^2^) and to have lower educational attainment. In addition, those individuals with hypertension were less likely to be current smokers or current drinkers, more likely to be former smokers, and were more likely to have a lower income and lower energy intake.

**Table 1:** Demographic Characteristics by Blood Pressure Status, (NHANES 1999–2006) (n = 12,440).

	Blood Pressure Status				
Participant Characteristic	Normotension (n = 4717)	Prehypertension(n = 3326)	Undiagnosed Hypertension	Diagnosed Hypertension(n = 3171)	P value**
Mean eGFR_MDRD_,					
mL/min/1.73 m^2^	91.9 (91.0–92.8)	88.5 (87.5–89.6)	83.4 (81.0–85.8)	82.0 (81.0–83.0)	<0.0001
Mean SBP, mm Hg	108.5 (108.2–108.8)	125.2 (124.9–125.6)	148.5 (147.3–149.7)	134.3 (133.4–135.1)	<0.0001
Mean DBP, mm Hg	66.7 (66.4–67.0)	74.9 (74.4–75.3)	81.4 (80.3–82.4)	75.3 (74.6–75.9)	<0.0001
Male	42.3 (40.6–44.0)	62.3 (60.1–64.5)	54.7 (51.3–58.0)	46.2 (43.9–48.5)	<.0001
Age at Screening, years	37.4 (36.9–38.0)	43.4 (42.7–44.0)	54.7 (53.3–56.1)	53.2 (52.3–54.1)	<0.0001
Race					
Mexican American	9.7 (8.2–11.3)	7.6 (6.1–9.1)	5.5 (3.8–7.3)	4.3 (3.1–5.4)	<0.0001
Other Hispanic	6.0 (4.3–7.8)	4.5 (2.7–6.3)	4.4 (2.1–6.6)	3.9 (2.6–5.2)	
Non-Hispanic White	71.1 (68.0–74.1)	73.0 (70.1–75.9)	74.2 (70.3–78.2)	75.8 (72.7–79.0)	
Non-Hispanic Black	8.6 (7.2–10.0)	10.1 (8.5–11.8)	11.2 (8.6–13.7)	12.2 (9.9–14.6)	
Other Race	4.6 (3.7–5.4)	4.7 (3.6–5.8)	4.7 (2.6–6.8)	3.7 (2.7–4.8)	
Educational Attainment					
Less Than High School	15.8 (14.2–17.4)	17.1 (15.5–18.7)	21.5 (18.9–24.2)	18.8 (17.0–20.6)	<0.0001
High School (including GED)	23.8 (21.9–25.8)	26.1 (24.3–27.8)	28.1 (25.4–30.8)	27.8 (25.5–30.0)	
Some College or Above	60.3 (57.7–63.0)	56.8 (54.5–59.1)	50.3 (46.7–54.0)	53.5 (50.9–56.1)	
Household income					
<$25,000	20.8 (18.9–22.7)	19.0 (17.2–20.8)	27.3 (23.7–30.9)	25.1 (23.1–27.0)	<0.0001
$25,000–$55,000	31.4 (28.9–34.0)	32.6 (30.6–34.6)	35.3 (31.2–39.4)	33.0 (30.4–35.5)	
>$55,000	47.7 (44.6–50.8)	48.4 (45.5–51.3)	37.4 (33.3–41.5)	42.0 (39.1–44.9)	
Body Mass Index, kg/m^2^	26.2 (25.9–26.4)	28.0 (27.7–28.2)	28.3 (27.8–28.8)	30.4 (30.0–30.7)	<0.0001
<30 kg/m^2^	19.8 (18.4–21.2)	29.8 (27.9–31.7)	32.4 (28.8–36.0)	44.0 (42.2–45.8)	<0.0001
≥30 kg/m^2^	80.2 (78.8–81.6)	70.2 (68.3–72.1)	67.6 (64.0–71.2)	56.0 (54.2–57.8)	
Cigarette Smoking Status					
Non–smoker	52.4 (50.4–54.5)	51.3 (48.7–53.9)	49.9 (46.7–53.1)	49.5 (47.5–51.5)	<0.0001
Former-smoker	16.3 (15.0–17.6)	19.8 (18.2–21.5)	27.5 (24.7–30.3)	28.8 (27.2–30.4)	
Current-smoker	31.3 (29.2–33.4)	28.9 (26.9–30.8)	22.6 (19.0–26.2)	21.7 (19.7–23.7)	
Alcohol Drinking Status					
Non-drinker	10.4 (8.5–12.4)	10.1 (8.0–12.3)	12.5 (9.1–15.9)	13.2 (11.0–15.4)	<0.0001
Former drinker	12.2 (11.1–13.4)	13.0 (11.2–14.9)	17.5 (15.0–20.1)	19.6 (17.6–21.7)	
Current drinker	77.3 (75.0–79.7)	76.8 (73.9–79.7)	70.0 (66.4–73.6)	67.2 (64.2–70.2)	
Daily Activities					
Sits, not walk very much	21.4 (19.9–22.8)	21.2 (19.4–23.0)	21.3 (18.0–24.5)	26.6 (24.8–28.4)	<0.0001
Walk, not carry much	50.3 (48.5–52.1)	48.3 (46.1–50.6)	54.3 (51.0–57.5)	51.2 (49.0–53.3)	
Light load	19.5 (18.0–20.9)	20.3 (18.6–22.0)	16.6 (14.2–18.9)	16.0 (14.4–17.5)	
Heavy work	8.8 (7.8–9.7)	10.2 (8.7–11.7)	7.9 (6.1–9.7)	6.3 (5.1–7.4)	
Dietary Intake					
Energy, kcal	2301.0 (2267.1–2334.9)	2427.0 (2377.0–2477.1)	2218.2 (2135.5–2300.9)	2126.9 (2079.3–2174.5)	<0.0001
ACR, mg/g	9.7 (8.7–10.6)	14.1 (11.4–16.9)	32.2 (23.4–41.1)	32.6 (22.8–42.4)	<0.0001
	5 (4–9)^#^	6 (4–10)^ #^	8.5 (5–18)^ #^	8 (5–16)^ #^	
<30	96.0 (95.3–96.7)	94.4 (93.4–95.3)	87.2 (85.4–89.1)	88.9 (87.7–90.2)	<0.0001
30–300	3.9 (3.2–4.6)	5.2 (4.3–6.2)	11.5 (9.6–13.3)	9.7 (8.5–10.9)	
>300	0.1 (0.0–0.2)	0.4 (0.2–0.6)	1.3 (0.7–1.9)	1.4 (0.9–1.9)	

Presented as weighted percentage % (95%CI,) or weighted mean (95%CI); ^#^Presented as Median (Q1–Q3);

* Rao-Scott chi-square test for categorical data, and survey regression model for continuous variables.

Abbreviations: eGFR, estimated glomerular filtration rate; SBP, systolic blood pressure; DBP, diastolic blood pressure;ACR, Albumin-creatinine ratios.

Shown in Table 2 is the age-adjusted prevalence of renal insufficiency by blood pressure status (normotension, prehypertension, undiagnosed hypertension, and diagnosed hypertension) and data from the further stratified analyses by albuminuria (urinary albumin-to-creatinine ratio (ACR)). The overall prevalence of eGFR_MDRD_ <90 mL/min/1.73 m^2^ was 63.2%, with prevalence of 54.07%, 2.35% and 0.06% for mild (eGFR 60–89 mL/min/m^2^), moderate (eGFR 30–59 mL/min/m^2^) and severe (eGFR<30 mL/min/m^2^) renal insufficiency, respectively. We found prevalence of mild renal insufficiency was not significantly associated with blood pressure status, whereas the prevalence of moderate-severe renal insufficiency moderately increased from normal subjects to prehypertensive, undiagnosed hypertensive and diagnosed hypertensive subjects (1.77%, 2.12%, 3.21%, 3.00%, respectively; *P* for trend, 0.0003). In the stratified analyses by level of albuminuria, we found among participants with ACR≥30 mg/g, the prevalence of moderate-severe renal insufficiency substantially and progressively increased from normal subjects to subjects with prehypertension, undiagnosed hypertension and diagnosed hypertension (1.43%, 3.44%, 10.96%, 11.70%, respectively; *P* for trend<0.0001). The prevalence of normal kidney function and mild renal insufficiency did not significantly differ by blood pressure status. On the other hand, there were no such trends among those with ACR<30 mg/g. We found very similar association patterns when the CKD-EPI equation was used (data not shown).

**Table 2:** Prevalence of Decreased Renal Function (eGFR_MDRD_) by Blood Pressure Status and stratified analyses by ACR, NHANES (1999–2006) (n = 12,440).

	Base on blood pressure measurement				
	Normotension	Prehypertension	Undiagnosed	Diagnosed Hypertension	P for trend
eGFR_MDRD_	N = 4,717	N = 3,326	N = 1,226	N = 3,171	
≥90, %	39.43(39.19–39.67)	38.35(38.14–38.57)	32.56(32.35–32.78)	36.43(36.20–36.66)	0.32
60 to 89, %	53.33(53.13–53.54)	53.36(53.18–53.53)	56.66(56.47–56.85)	53.35(53.15–53.54)	0.58
<60, %	1.77(1.73–1.81)	2.12(2.08–2.16)	3.21(3.14–3.27)	3.00(2.94–3.06)	0.0003
45–59, %	1.78(1.75–1.82)	2.04(2.00–2.08)	2.90(2.84–2.96)	2.62(2.57–2.68)	0.04
30–44, %	0.05(0.04–0.05)	0.07(0.07–0.08)	0.16(0.15–0.17)	0.17(0.16–0.19)	0.007
<30, %	0.02(0.02–0.03)	0.04(0.04–0.05)	0.09(0.08–0.10)	0.14(0.13–0.16)	0.07
ACR≥30	N = 204	N = 211	N = 199	N = 453	
eGFR≥90, %	48.35(47.15–49.55)	39.62(38.70–40.55)	24.81(23.95–25.68)	29.49(28.60–30.39)	0.36
eGFR 60–89, %	46.49(45.38–47.59)	48.01(47.13–48.89)	53.17(52.16–54.18)	49.84(48.88–50.80)	0.84
eGFR <60, %	1.43(1.30–1.58)	3.44(3.19–3.70)	10.96(10.27–11.68)	11.70(11.06–12.36)	<0.0001
ACR<30	N = 4,513	N = 3,115	N = 1,027	N = 2,718	
eGFR≥90, %	39.06(38.81–39.30)	38.00(37.79–38.22)	31.50(31.29–31.72)	35.75(35.51–35.98)	0.38
eGFR 60–89, %	53.91(53.70–54.13)	54.15(53.96–54.34)	58.37(58.17–58.57)	54.78(54.57–54.99)	0.88
eGFR <60, %	1.69(1.65–1.73)	1.92(1.88–1.96)	2.87(2.80–2.94)	2.49(2.43–2.56)	0.06

Prevalence estimates of kidney disease (%) were adjusted for age, sex and race/ethnicity in individual logistic regression models.

Abbreviations: eGFR, estimated glomerular filtration rate, mL/min/1.73 m^2^;ACR, Albumin-creatinine ratios, mg/g.

We also did stratified analyses of age-standardized prevalence of different blood pressure status (normotension, prehypertension, undiagnosed hypertension, and diagnosed hypertension) by albuminuria (data not shown). The overall prevalence of prehypertension, undiagnosed hypertension and diagnosed hypertension was 26.74%, 9.86% and 25.49%, respectively, and remained similar among the 3 GFR stages (eGFR_MDRD_≥90 mL/min/1.73 m^2^, 60–89 mL/min/1.73 m^2^, and <60 mL/min/1.73 m^2^). In stratified analyses by ACR, we found the prevalence of undiagnosed hypertension was much higher in those with albuminuria compared to those without albuminuria (25.82% vs. 13.57% for those with normal renal function, 19.09% vs. 13.36% for those with mild renal dysfunction and 35.54% vs. 12.62% for those with moderate-severe renal dysfunction, *P* value <0.05), whereas the prevalence of prehypertension was similar. Again, similar findings were observed when the CKD-EPI equation was used (data not shown).

## Discussion

Worldwide, hypertension is one of the most common chronic diseases [4] while a ‘silent’ epidemic of CKD has also developed in recent years [17]. In a nationally representative study population, we found even among adults without known CKD, diabetes or cardiovascular diseases, the prevalence of reduced renal function, prehypertension and undiagnosed hypertension was still high (63.2%, 26.74% and 9.86% respectively). Furthermore, we found prehypertension or undiagnosed hypertension was associated with reduced kidney function only among those with albuminuria (ACR≥30 mg/g), but not among those without.

Strict blood pressure (BP) control has been considered the basis of therapy for slowing renal deterioration [18]. However, very recently, the follow-up cohort study for the AASK trial [16] showed that among African Americans, intensive BP control had no overall effect on CKD progression, but there was a potential benefit in patients with albuminuria. A pooled analysis showed that a lower BP goal might delay decline in GFR among patients with a greater urine protein excretion [19]. The underlying mechanism remains unknown. However, a number of previous studies [20]–[22] have shown that not only albuminuria levels but also albuminuria changes can be used to predict cardiovascular and renal outcomes. Even among the patients with a so-called normal threshold of microalbuminuria, an increased risk of total mortality and cardiovascular and renal events in patients with albuminuria between 10 and 30 mg/g creatinine was observed as compared with the patients with albuminuria less than 10 mg/g. The follow-up cohort study for MDRD trial did not find the modification effect by albuminuria as shown in the initial trial. It is possible that the inconsistent result is because more participants in the intensive BP control group used angiotensin-converting-enzyme (ACE) inhibitors in the MDRD study [23].

The association between high blood pressure and renal insufficiency is complex and multifactorial. It is believed hypertension and CKD may mutually be both the cause and consequence of each other [24], [25]. A causal relationship is hard to draw from previous human studies investigating the association [26]–[28]. Furthermore, previous studies may be confounded by medication use for hypertension or CKD or dietary changes after diagnosis. A recent study examined the association between prehypertension and known CKD [29]. However, the patients with CKD may change their diets or take medications. The current study evaluated the association of prehypertension or undiagnosed hypertension with mildly reduced kidney function or undiagnosed CKD among adults without diabetes, CKD or cardiovascular disease. Thus, our finding may help to understand the relationship without interference from medication use for blood pressure control and diet changes after diagnosis.

We found the association between high blood pressure and moderate-severe renal insufficiency only appeared statistically significant among those with albuminuria (ACR≥30 mg/g), but not among those with ACR<30 mg/g. This finding suggests kidney function may be associated with elevations in blood pressure only when albuminuria is present. This might provide a possible explanation for the renal-protective effects of more aggressive BP control only occurring among participants with albuminuria in previous large-scale clinical trial studies [12], [16]. Albuminuria may play a crucial pathogenetic role,serving as a surrogate marker of increased glomerular capillary pressure and a predictor of progressive renal function loss [30]. In the setting of proteinuria, lower eGFR indicates clinically significant renal injury and higher risks for both cardiovascular and renal endpoints [31], and is reasonably associated with hypertension. On the other hand, our finding that when albuminuria is present hypertension is also more likely to be associated with moderate-severe reduction in GFR may reflect a common clinical phenomenon for later stages of CKD progression [32]; and this finding is in agreement with previous clinical trials [12]–[14], [16], [19]. However, all previous trials were conducted exclusively among CKD patients with a moderately to severely decreased GFR. The findings from the current study suggest intensive control of blood pressure might be of additional benefit among those possessing albuminuria, but only a mildly reduced or normal kidney function. Further studies are necessary to investigate these issues and understand the detailed mechanisms.

The current study has several strengths and implications. This is a population-based study with nationally representative samples. Using eight years of NHANES data provides a large sample and precise estimates of undetected chronic conditions. Participation rates for the interview and medical examination center during 1999–2006 NHANES were high, which may minimize potential selection biases. We have excluded participants with self-reported kidney diseases,diabetes or cardiovascular diseases in the analyses to minimize the influence of diet change or medication use on blood pressure, urinary albumin excretion or renal function. We have also examined the associations using both the MDRD and CKD-EPI equations and found similar results. However, as with all cross-sectional studies, one major concern is that the temporal sequence may not be clear. It is possible that our results could be due to reverse causation. Findings of this study should be considered preliminary. Thus, future prospective studies are necessary to further replicate our findings. It is possible that albuminuria underlies the association between high blood pressure and renal dysfunction [33], [34]. However, it is also possible that the coexistence of hypertension, albuminuria and decreased GFR is in response to the same underlying cause (e.g. physically inactive life style, stress), and therefore in this regard, our study design may not be well-suited to evaluate their relative contributions., Also, the number of subjects with elevated albuminuria was relatively low (n = 1067). Additionally, nondifferential misclassification may occur because there was only one measurement of creatinine concentration or urinary protein. However, nondifferential misclassifications usually bias the results to the null and, thus, our estimates would be conservative. Although we have found a significant overall association between high blood pressure and renal function insufficiency in all participants analyzed which is very similar to a recent study using the NHANES dataset, there are some discrepancies on prevalence rates of pre- and hypertensive subjects. It is likely that the differences may be primarily because we excluded those who self-reported kidney diseases, diabetes or cardiovascular diseases in the current analyses while the previous study did not.

In conclusion, our cross-sectional study found the association between high blood pressure and reduced renal function was modified by the albuminuria status among participants without diabetes, CKD or cardiovascular disease, excluding interference from medication use or dietary changes. Furthermore, our findings suggest this also could be true among those with a mildly reduced or normal kidney function. Further studies are warranted to confirm our findings and explore this promising possibility.

## Methods

### Study Population

The NHANES consists of cross-sectional, multistage, stratified, clustered probability samples of the civilian non-institutionalized US population [35]. The surveys are conducted by the National Center for Health Statistics, including a standardized in-home interview and a physical examination and blood and urine collection at a mobile examination center. The data are appropriate for estimating the prevalence of chronic conditions in the United States. In 1999, NHANES became a continuous annual survey to allow annual estimates, with release of public-use data files every 2 years. The 1999–2006 NHANES data files included 41,474 participants.

Our study was limited to adult participants from 1999–2006 NHANES, who were 20 years or older (n = 20,311) and were not pregnant or lactating (n = 18,992). Because the presence of chronic diseases may affect renal function, we excluded those with self-reported kidney disease (18421), or cardiovascular diseases (16207), or diabetes, (n = 14,717). Also excluded were those with missing values for blood pressure, urine protein excretion, or serum creatinine concentration. As a result, 12,440 adult participants were included in the final analyses.

### Measurements

A standardized protocol for measurements of auscultatory blood pressure (BP) was executed during the examination center visit by a trained physician using a mercury sphygmomanometer with appropriate cuff sizes. Recorded systolic BP (SBP) and diastolic BP (DBP) were the means of three (sometimes four) measurements separated by 30 seconds after a 5-minute rest period [36]. The averages of all of the available measurements for systolic and diastolic blood pressure were used in our study.

Self-reported information on demographics (age, sex, and race), socioeconomic status (education, insurance, and income), and lifestyle factors (smoking, drinking, physical inactivity and obesity) were obtained during the interview portions of the surveys. Height and weight were measured at the examination center. Random spot urine samples were obtained. Urine albumin was measured using solid-phase fluorescence immunoassay. For those with albuminuria measurement lower than the assay’s detection limit, 0.2 µg/ml was assigned [37]. Urine creatinine was measured using the modified Jaffe kinetic method, and serum creatinine was measured by a kinetic rate Jaffe method using different analyzers in different survey years [38].

### Definitions

We classified the participants into the following four BP categories: 1) self-reported hypertension are those who answered ‘yes’ to the question ‘Have you ever been told by a doctor or other health professional that you have hypertension, also called high blood pressure?’. Among those who answered ‘no’ to that question, we defined them based on their measured averaged systolic or diastolic blood pressure: 2) participants who had undiagnosed hypertension are those who had either measured SBP≥140 mm Hg or DBP≥90 mm Hg; 3) participants who had prehypertension are those whose SBP ranged from 120–139 mmHg or DBP ranged from 80–89 mmHg; and 4) participants who had normotension are those who had SBP<120 mmHg and DBP<80 mmHg, according to the Seventh Report of the Joint National Committee on Prevention, Detection, Evaluation, and Treatment of High Blood Pressure (JNC 7) [2]. Self-reported kidney diseases are those who answered ‘yes’ to the question ‘Have you ever been told by a doctor or other health professional that you had weak or failing kidneys? Do not include kidney stones, bladder infections, or incontinence.’

To appropriately estimate glomerular filtration rate (GFR), all serum creatinine measurements were re-calibrated to standardized creatinine measurements obtained at the Cleveland Clinic Research Laboratory following National Kidney Disease Education Program (NKDEP) [39] and NHANES [40] recommendations. Serum creatinine-based GFR was estimated using the 4-variable Modification of Diet in Renal Disease (MDRD) Study equation: eGFR (mL/min/m^2^) = 175.0× (SCr)^−1.154^×age^−0.203^×0.742 (if female) ×1.212 (if African American), where age was expressed in years and SCr was standardized serum creatinine level in milligrams per deciliter [41]. We also conducted analyses using eGFR calculated with the newly developed Chronic Kidney Disease Epidemiology Collaboration (CKD-EPI) equation [42]. We classified the participants into the following three GFR categories: 1) normal renal function with eGFR≥90 mL/min/m^2^; 2) mildly decreased renal function with eGFR 60–89 mL/min/m^2^; and 3)moderately to severely decreased renal function with eGFR<60 mL/min/m^2^ [43], [44].

Urinary albumin-to-creatinine ratio (ACR) was computed in milligrams per gram. Albuminuria was defined as ACR≥30 mg/g, consistent with the position statement of the National Kidney Foundation and the National Institute of Diabetes and Digestive and Kidney Diseases [45].

### Statistical analyses

We performed statistical analyses using the Survey procedure in SAS 9.2 software (SAS Institute, Cary, NC) to estimate variance after incorporating the weights for the sample population, which took into account unequal selection probabilities and planned oversampling of certain subgroups resulting from the complex NHANES multistage stratified cluster sample design [44]. 8-year weights were used in our analysis which calculated as follows: 4-year weight/2 (for NHANES 1999–2002); or 2-year weight/4 (for NHANES 2003–2004, NHANES2005–2006). Covariates were compared between BP groups to evaluate potential confounding factors using Rao-Scott chi-square test for categorical data and survey regression model for continuous variables. To derive estimates of the prevalence of renal insufficiency for each blood pressure group, survey logistic regression model was performed adjusted for age (continuous), sex and race/ethnicity using the 2006 US Census estimates for the >20 years old US population [46]. All of the reported P values were two-sided with statistical significance evaluated at 0.05.
